# Authentic leadership and flourishing: Do trust in the organization and organizational support matter during times of uncertainty?

**DOI:** 10.3389/fpsyg.2022.955300

**Published:** 2022-09-06

**Authors:** Deon J. Kleynhans, Marita M. Heyns, Marius W. Stander

**Affiliations:** Optentia Research Entity, North-West University, Vanderbijlpark, South Africa

**Keywords:** authentic leadership, flourishing, trust in the organization, organizational support, precariousness

## Abstract

**Orientation:** This study investigated the influence of authentic leadership on employee flourishing while considering the potential mediating effect of trust in the organization and organizational support as underlying mechanisms in an uncertain setting.

**Research purpose:** To examine the relationship between authentic leadership and employee flourishing by evaluating the indirect effect of organizational support and trust in the organization as potential mediators.

**Motivation for the study:** An authentic leadership approach, organizational support, and trust in the organization may influence the flourishing of employees in uncertain times. Increasing the comprehension of the possible interaction effect of organizational support and trust in the organization in the relationship between authentic leadership and employee flourishing may improve individual and organizational efficiency.

**Research approach/design and method:** A quantitative, cross-sectional survey design was applied in this study. The sample comprised 314 employees in a noteworthy South African steel manufacturing entity. The Authentic Leadership Inventory, Workplace Trust Survey, Flourishing-at-Work Scale, and the Job Demands-Resources Scale were administered.

**Main findings:** The findings of this study suggest that authentic leadership was a significant predictor of employee flourishing through organizational support and trust in the organization.

**Practical/managerial implications:** This research illuminates the potential value-adding contribution of an authentic leadership style in promoting a trust-filled relationship between team members and their organization and the support they experience from their employer. Despite the prevailing precarious context, working under the mentioned conditions might result in the increased flourishing of employees.

**Contribution/value-add:** The analyses of the mentioned relationships might assist businesses in optimizing the resources required to improve employee and organizational performance. Additionally, the exploration of organizational support in conjunction with organizational trust raises our understanding of the possible influence these elements can have in enhancing employee flourishing in the workplace.

## Introduction

The 21*^st^*-century international economic climate has been characterized by organizational pressures and challenges in order to adhere to stakeholder requirements (Hameed and Sharma, 2020). Apart from the other difficulties that business entities have faced, the outbreak of the COVID-19 pandemic brought additional challenges that needed to be navigated. COVID-19 (also called the coronavirus) was declared a global disease outbreak of worldwide concern on 30 January 2020 and then a pandemic on 11 March 2020 (World Health Organization [WHO], 2020b). With more than 200 countries and territories as well as most organizations being affected by around 441 million cases and fatalities exceeding five million (Worldometer, 2022), the COVID-19 pandemic continues to be one of the most significant global crises of the modern era (World Health Organization [WHO], 2020a). Its worldwide effect is visible in the more than 157 million cases in Europe, over 149 million in the United States, more than 118 million in Asia, and over 11 million in Africa (Worldometer, 2022). Despite the increasing number of COVID-19-related research studies, organizations are troubled by the uncertainties accompanying the pandemic (Gostin and Wiley, 2020). Governing bodies, policymakers, and researchers are increasingly concerned about the adverse effect that COVID-19 can have on business management practices and the international economy (Amankwah-Amoah, 2020).

The influence of the ongoing global health dilemma, together with the international efforts to try and contain the virus, has not only affected the worldwide economy and human health, but also complicated the careers and working life of countless individuals (Restubog et al., 2020). The pandemic has brought about abrupt changes in the workplace (e.g., remote working and virtual teamwork), while affecting the work-life of employees (e.g., social distancing, anxiety, and job insecurity) (Kniffin et al., 2021). The anxiety of individuals has, furthermore, been kindled by profoundly emotional messages, disturbing images, and broadly reported death tolls (Peters, 2020).

Additionally, the pandemic and the resulting changes may have resulted in workplace-related uncertainty due to followers feeling concerned about the future of their employer, their working conditions, and the security of their jobs (Chirumbolo et al., 2021; Obrenovic et al., 2021). While being in an employment relationship during times of uncertainty, the unpredictability and precariousness may still lead to employees experiencing anxiety and stress (Ererdi et al., 2021; Greyling et al., 2021) that can adversely influence their emotional and psychological well-being in the short term and can cause burnout over the medium to long term (Dewey et al., 2020). In support of previous research suggesting that an uncertain and insecure work context has a negative impact on team member health and well-being (Standing, 2011; Utzet et al., 2020), recent studies have emphasized the adverse effects of COVID-19 on job satisfaction, well-being (Bakker and Van Wingerden, 2021), and social relations (Möhring et al., 2021). Moreover, the International Labor Organization mentioned that the costs related to psychological health complications are equivalent to 3–4% of the European Union’s gross domestic product (Organisation for Economic Co-operation and Development, 2012). Chisholm et al. (2016) posit that psychological health difficulties result in an annual loss in productivity of an estimated $925 billion. When considering these costs, a clear need exists for research on the identification of psychological health predictors (Caesens et al., 2021). Because employees spend many hours at work, the way employers treat and value their employees has a prominent effect on their well-being, both inside and outside of the workplace (Eisenberger and Stinglhamber, 2011).

Huppert and So (2013) state that hedonic and eudaimonic well-being are components of employee flourishing and that flourishing employees are able to handle uncertainties and challenges more effectively than those who are not flourishing (Schotanus-Dijkstra et al., 2016). The importance of follower well-being is reiterated in a study by Krekel et al. (2019), which suggests that employee well-being is positively linked to organizational performance and team member productivity. Safeguarding the well-being of employees has, thus, become a primary focus area for most business owners during the pandemic (Deloitte, 2020). This study extends the existing knowledge base by developing a more nuanced understanding of selected factors that may affect flourishing as these manifests within a post-COVID work environment characterized by uncertainty and volatility.

Amid uncertain and challenging conditions, such as the COVID-19 period, the role of leaders is imperative (Baran and Woznyj, 2020; Rath et al., 2021), as followers depend on them for support and guidance. During COVID-19 the role of the leader changed from face-to-face interaction to having virtual interactions, limiting the time spent on one-to-one interactions and opportunities to support employees. Farhan and Wright (2021) stressed the importance of a leadership model that create a culture of care and trust in uncertain times. According to Wang et al. (2014), authentic leadership is a positive leadership style that can be described as a transparent, moral, and genuine approach that can make a constructive contribution during periods of precariousness. Although authentic leadership has generated interest among researchers, there seems to be limited research involving the relationship between authentic leadership and employee well-being (Inceoglu et al., 2018), especially in volatile times. Authentic leaders have the ability to positively affect team member trust in the organization, intrinsic motivation, job satisfaction, and commitment (Miniotaite and Buciuniene, 2013; Tabak et al., 2013) and may add value in times of uncertainty. Furthermore, Yijia and Jinhong (2016) maintain that authentic leadership is positively associated with perceived organizational support. Imran et al. (2020) found perceived organizational support to be linked positively to flourishing and work engagement. Flourishing, trust in the organization, and career progression were also found to be associated with perceived trust in the workplace (Caesens et al., 2017). Rautenbach and Rothmann (2017b) indicate that organizations can benefit when promoting authentic leadership, as the flourishing of their employees will be enhanced.

Tuzovic and Kabadayi (2021) posit that, while endeavoring to identify the factors that may have an impact on employee well-being, researchers have applied the job demands-resources (JD-R) model (Bakker and Demerouti, 2007) in many well-being-related studies. Subsequently, the JD-R model was also used in this study. According to the principles of the JD-R model, having applicable job resources available when job demands are high will benefit the organization (Bakker et al., 2007). To this end, applying the JD-R model approach could be to the advantage of the entity where this study was conducted, as it was functioning in challenging circumstances. Outside forces, including technology, certain factors in the business environment, and government regulations, may affect job demand and resource levels (Bakker et al., 2003). When the mentioned external factors change, it may result in a change in the demands and resources of employees, which can influence their well-being (Brauchli et al., 2013). Due to the possibility that workplace well-being can increase when the job resources at team members’ disposal may capacitate them to reduce the impact of job demands (Bakker et al., 2014), it may be advantageous to find and apply identified job resources to mitigate the effect of job demands (Lesener et al., 2019).

Very little, if any, research is available on these specific constructs during times of uncertainty, in this case during a global pandemic. The central research question that can be asked is whether authentic leadership will positively influence employee attitudes in times of uncertainty? We know that authentic leadership is a positive predictor of organizational support (Aria et al., 2019; Baykal, 2020), trust in the organization (Stander et al., 2015; Coxen et al., 2016), and flourishing (Chevalier et al., 2021; Kleynhans et al., 2022). However, it is not clear to what extend the impact on each other play in a model incorporating all the constructs. Consequently, the link between perceived organizational support and organizational trust as serial mediators between authentic leadership and flourishing needs more investigation. Considering the gaps illustrated above, the aim of the paper is to reveal the linkage between authentic leadership, organizational support, trust in the organization, and flourishing in uncertain times. The paper seeks to answer the following: (a) What is the potential value of authentic leadership in the way employees experience organizational support, trust in the organization, and flourishing during times of uncertainty? (b) Will authentic leadership strengthen employees’ perceptions of organizational support, thereby enhancing their trust in the organization and, consequently, furthering higher levels of flourishing?

This paper aims to make three main contributions to the existing body of knowledge. First, it intends to enrich the leadership literature by identifying how authentic leadership affects work-related attitudes in terms of the experience of organizational support, leading to trust in the organization, and flourishing of employees in uncertain times. Second, the paper deals with the chain of effects between authentic leadership, organizational support, trust in the organization, and flourishing and captures the expected positive effect caused by authentic leadership. Third, practical implications for leaders will be discussed.

## Literature review and hypothesis

### The relationship between authentic leadership and organizational support, trust in the organization, and employee flourishing

The notion of flourishing has become one of the most notable multifaceted well-being-related models (Keyes, 2002; Seligman, 2011). Flourishing consists of two components, namely, hedonic and eudaimonic wellness. Hedonic wellness refers to positive emotions and a life filled with good feelings, while eudaimonic wellness relates to how individuals nurture their abilities to function well in their everyday life (Keyes, 2006). Keyes and Annas (2009) state that flourishing encompasses the subjective well-being of persons, involving psychological, emotional, and social features.

Flourishing in a work context can be separated into flourishing, mental well-being, and languishing (Rautenbach and Rothmann, 2017a). A person’s mental health state can be measured across a continuum. Individuals who flourish are plotted at the one end of the continuum and those who languish at the other end (Keyes, 2002). As can be deduced, those with a positive state of mental health are at the higher end of the spectrum and deemed to flourish. Employees who flourish at work feel well (i.e., they are content with their job and have positive emotions, perform psychologically well) (i.e., are devoted and determined and find purpose at work), and are socially well-adapted (i.e., are socially accepted, make social contributions, and are socially growing) (Rothmann, 2013). Individuals who are in a languishing state of being are at the lower end of the mental health spectrum. According to Keyes (2006), most individuals experience a moderate mental health state and are located between a languishing and a flourishing state on the spectrum. The well-being of individuals is not only viewed as a desirable end state in psychological terms but improving the well-being of employees is also regarded as beneficial for business and, thus, something to aspire to (Schotanus-Dijkstra et al., 2016). The contribution that well-being can make in the workplace is demonstrated by findings that it relates to work engagement (Diener et al., 2003; Bakker and Oerlemans, 2011; Ariza-Montes et al., 2018), career development (Rosenbaum et al., 2011), hope for the future (Pleeging et al., 2021), and improved productivity (Oswald et al., 2015). Authentic leaders are likely to cultivate positive social exchanges, they can have a positive effect on team member well-being, as positive social exchanges enhance well-being (Ilies et al., 2005).

According to Rego et al. (2016), authentic leadership originated from the constructive perspective and action of leaders who positively influenced their followers. Authentic leadership grants a framework of positive, trusting, and ethical leadership (Kiersch and Peters, 2017). Even though authentic leadership has been described in numerous ways and analyzed in several studies (Gardner et al., 2011), the definition most often employed is the description proposed by Walumbwa et al. (2008): “A pattern of leader behavior that draws upon and promotes both positive psychological capacities and a positive ethical climate to foster greater self-awareness, an internalized moral perspective, balanced processing of information, and relational transparency on the part of leaders working with followers, fostering positive self-development” (p. 94).

According to the description, authentic leadership consists of four dimensions, namely, self-awareness, internalized moral perspective, balanced processing, and relational transparency (Banks et al., 2016). Self-awareness relates to the awareness of, and trust in, an individual’s values, cognitions, and motives. Internalized moral perspective points to behaving in accordance with one’s true self rather than displaying behavior directed at gaining the favor of external parties or avoiding some form of punishment. Balanced processing pertains to the unbiased processing of available information that includes relevant knowledge during decision-making without contradicting, manipulating, or overstating available evidence. Lastly, relational transparency refers to the self-disclosure of an individual, the person’s genuine convictions and emotions, and the establishment of trust-based associations. Authentic leadership is an original, ethical, and transparent leadership style known for its positive approach during times of uncertainty (Wang et al., 2014). When displaying authentic leadership behavior, such as a positive moral perspective, elevated awareness, and timely and clear communication, leaders can inspire team members to achieve work-related goals (Crawford et al., 2020).

Authentic leadership was also found to predict organizational support (Vermeulen, 2017; Gojny-Zbierowska, 2018; Aria et al., 2019). According to Eisenberger et al. (1986), perceived organizational support is defined as “employees in an organization form global beliefs concerning the extent to which the organization values their contributions and cares about their well-being” (p. 500). However, organizations have different ways of expressing to its employees that it values their contribution and that it cares for their well-being (Kurtessis et al., 2017). One way of demonstrating to employees that the organization cares about their well-being is through its leaders because they can influence the connection that employees feel toward the organization (Kurtessis et al., 2017). To this end, Cropanzano et al. (2001) determined that fairness and an inspirational and supportive leadership style is a strong predictor of perceived organizational support. The results from a recent study conducted by Kurian and Nafukho (2022) suggest that authentic leader can create high levels of perceived fairness due to the climate they create for their team members. Moreover, authentic leaders might have a positive effect on employee behavior due to the support and assistance that they provide to employees to develop and perform at their best (Ilies et al., 2005). Lastly, because authentic leaders are self-aware, have high moral standard and build transparent relationships, they can build positive relationships with followers, while inspiring and encouraging them to achieve their goals (Deshwal and Ali, 2020). It is thus plausible to suggest that authentic leadership associates with organizational support.

A study conducted by Chen and Sriphon (2022) indicated that authentic leadership was linked positively to both social exchange relationships and trust. Furthermore, an authentic leadership style can promote a healthy and supportive work setting characterized by sound relationships and trust among team members, follower trust in their leaders (Asad et al., 2021), and in their organization (Miniotaite and Buciuniene, 2013; Alkaabi, 2018; Kleynhans et al., 2021a). It is thus plausible to argue that increased levels of trust can be ascribed to the example set by authentic leaders who are characterized by high moral standards, honesty, transparency, and integrity.

Recent studies found that authentic leadership could be beneficial to organizations, as it was associated with a variety of positive end results, such as follower creativity, team member performance (Duarte et al., 2021), consumer focus, personnel retention (Ribeiro et al., 2020), and employee flourishing (Nair et al., 2021; Kleynhans et al., 2022). Ryff and Keyes (1995) maintain that when leaders exhibit behavior linked to the dimensions of authentic leadership (self-awareness, internalized moral perspective, balanced processing, and relational transparency), they can influence the well-being of employees. For example, self-awareness and balanced processing could assist followers with increases self-acceptance, personal growth, and the mastery of their environment, while relational transparency should lead to positive relationships in the workplace.

Considering the information provided above, it can be argued that an authentic leadership approach can have a positive influence on employees’ perceptions regarding the support provided by the organization, their trust in the organization, and their levels of flourishing. Accordingly, the following hypothesis was formulated:

**Hypothesis 1:** authentic leadership positively influences (a) organizational support, (b) trust in the organization, and (c) employee flourishing.

### Organizational support as a mediator in the relationship between authentic leadership and employee flourishing

Organizational support theory was introduced in 1986 by the American social psychologist Eisenberger, who maintained that perceived organizational support was represented by follower perception of the value that an organization attached to followers’ contributions and the care it demonstrated for their well-being (Eisenberger et al., 1986; Sun, 2019). When applying the social exchange theory, perceived organizational support implies that followers provide the employing organization with their capabilities, time, attitude, and efforts for favorable treatment from the employer (remuneration, development, career advancement, support, etc.) (Kurtessis et al., 2017). The support that organizations provide to their employees assists in meeting their own needs and demonstrates their willingness to supply the employee with material and other support.

Based on the principle of reciprocity, how organizations treat their employees can influence perceived organizational support, which, in turn, will have an impact on employee attitudes and behavior (Sun, 2019). When team members, thus, perceive that the company has their best interests at heart, demonstrated by its commitment and support, they are likely to reciprocate by showing loyalty and adopting the actions and attitudes required by the company (Sun, 2019). Examples of the support organizations can provide to their employees include how they react to employee health issues, the errors that employees make, and their performance (good or bad), their provision of meaningful and challenging work, the provision of employee benefits, and payment of a fair wage (Sun, 2019).

Authentic leaders can influence the climate within an organization (Woolley et al., 2011) and the well-being of team members by means of different mechanisms (Ilies et al., 2005). Because of their open and honest communication, and the trust and sincere interest these leaders have in their team members (Gardner et al., 2005), they cultivate a positive and safe climate where followers can feel free to express themselves (Nelson et al., 2014). The perception of employees regarding the support provided by their leader, who is considered by them as a representative of the organization, is hereby positively influenced resulting in them extending that perception to the organization.

Previous research indicated that perceived organizational support can be linked to the enhancement of job satisfaction and flourishing while reducing the stress levels of employees (Kurtessis et al., 2017). According to Ho and Chan (2022), and in line with the organizational support theory (Kurtessis et al., 2017), perceived organizational support was identified as a prominent contributor to employee flourishing. These findings imply that when followers perceive that their organization or their leader appreciates their contribution, values their well-being, and demonstrates support, these employees will likely experience increased levels of flourishing. Based on this discussion the following hypothesis was formulated:

**Hypothesis 2:** organizational support mediates the relationship between authentic leadership and employee flourishing.

### Trust in the organization as a facilitative mechanism in the relationship between authentic leadership and employee flourishing

Trust is an essential aspect of any human relationship, and although there seems to be no unanimously accepted description of trust, scholars tend to agree on the key defining features that describe trust. Accordingly, Rousseau et al. (1998) posit that trust can be described as a psychological state that consists of “the intention to accept vulnerability based upon positive expectations of the intention or behavior of the other” (p. 395). Trust includes an element of risk perception (Siegrist et al., 2021), while being a future-oriented state shaped by the actions and behavior of another party (Corbett and Le Dantec, 2018). In an organizational context, trust can be classified as interpersonal and impersonal. Examples of interpersonal trust are trust in colleagues and in leaders, an example of impersonal trust is trust in the organization (Güçer and Demirdağ, 2014; Haynes et al., 2020; Oliveira et al., 2020). When considering previous research studies, trust in leaders has frequently been investigated (Ullah et al., 2019) and trust in colleagues somewhat less (Bachmann et al., 2015). Trust in the organization is, however, a relatively new phenomenon that requires more in-depth analysis (Jaškevičiūtė, 2021) and is one of the constructs used in this study.

When trust in the workplace is diminished, it can function as a barrier, resulting in increased vulnerability, heightened caution among employees (which leads to diminished levels of effort), and, thus, reduced benefits flowing from team member efficacy (Bandura, 2001; Dirks and Ferrin, 2001). To this end, trust in the organization is represented by the trust stakeholders (including employees) have in an entity and includes perceived factors such as the fairness and transparency of actions, processes, and procedures (Rawlins, 2008; Schnackenberg and Tomlinson, 2014). Additionally, the way companies are true to their word also plays a vital role in perceived trust. Trust in the organization refers to a setting where positive attitudes/viewpoints, elevated performance, and collaboration are highly likely (Brown et al., 2014). Trust in the organization and trust in its leaders were found to associate with vital outcomes, which included organizational commitment (Baştuğ et al., 2016), organizational citizenship behavior (Singh and Srivastava, 2016; Dhiman and Sharma, 2021), employee performance (Ekhsan and Saroh, 2021), knowledge management and organizational performance (Fitria, 2020), and employee well-being (Jaškevičiūtė, 2021).

Research indicates that a trusting relationship is established through the social exchange between parties (Wang et al., 2019). The social exchange theory involves the processes and principles related to the exchange of treasured social and psychological commodities (Cross and Dundon, 2019). A key concept of social exchange theory is that participants in a relationship feel obliged to reciprocate the commodities at their disposal because they are inspired to safeguard the balance between inputs and outputs to preserve a social transaction equilibrium (Buchanan, 2021).

The prevalence of trust in the organization can have an impact on work-related outcomes by influencing how employees appraise the anticipated behavior of the business (Dirks and Ferrin, 2001; Adnan et al., 2018). When the future actions of an organization are perceived positively by employees, the resulting trust in the organization can lessen the negative impact of the uncertainty that may prevail (Dirks and Ferrin, 2001), having a positive impact on employee outcomes such as well-being. Research studies determined, among others, that perceived trust in the organization was positively associated with employee well-being (Kurtessis et al., 2017; Imran et al., 2020; Ho and Chan, 2022), employee retention (Baranchenko et al., 2020), job satisfaction (Andriyanti and Supartha, 2021), employee performance (Shabbir et al., 2021), and organizational performance (Imran and Aldaas, 2020). Conversely, when employees perceive that they are treated unfairly by the organization, their confidence and trust in the organization will likely suffer, leading emotional exhaustion and reduced employee well-being.

According to Kleynhans et al. (2021a), there is a positive relationship between trust in the organization and authentic leadership, while Paolini et al. (2020) found an association between trust and well-being during times of uncertainty. Because authentic leaders can be described as those “who are deeply aware of how they think and behave and are perceived by others as being aware of their own and others values/moral perspectives, knowledge and strengths; aware of the context in which they operate; and who are confident, hopeful, optimistic, resilient, and of high moral character” (Avolio et al., 2004, p. 4), it can be argued that authentic leadership associates with trust and the well-being of employees. Additionally, the possible mediating effect of trust in the leader on the relationship between authentic leadership and the attitudes and behaviors of employees in the workplace has been confirmed in previous studies (Wang and Hsieh, 2013; Agote et al., 2016). The following hypothesis was thus formulated:

**Hypothesis 3:** trust in the organization mediates the relationship between authentic leadership and employee flourishing.

### Testing the simultaneous, serial mediating effect from organizational support to trust in the organization in the relationship between authentic leadership and flourishing

This study investigated the relation between authentic leadership, Flourishing, organizational support, and trust in the organization during times of uncertainty.

While the prevalent challenges in the world economy, society, and the labor environment have resulted in the occupational environment being increasingly precarious, the outbreak of the COVID-19 pandemic worsened the situation, putting the health and well-being of individuals at risk (Giunchi et al., 2019; Alradhawi et al., 2020; Jajodia et al., 2020; Rajendran et al., 2020). Many business entities were affected negatively by the outbreak of the coronavirus pandemic and were forced to implement measures to contain the spread of the virus, while adversely affecting their business processes and outcomes (Donthu and Gustafsson, 2020; Sohrabi et al., 2020). Because individuals had to start practicing social distancing and self-isolation and limit their travels to try and contain the spread of the virus, it affected organizational activities (Nicola et al., 2020) and possibly the well-being of individuals negatively.

Drawing on the JD-R model, followers require appropriate resources that will equip them to cope with challenges in the workplace (Bakker and Demerouti, 2007; Schaufeli, 2017). The well-being of employees is likely to improve if they have adequate resources at their disposal to face taxing job demands. In contrast, when employees are confronted with high work demands and have insufficient resources, it may have a negative impact on their well-being.

Many researchers support the notion that effective leadership is vital in times of uncertainty and insecurity because the organization and its followers need the support and encouragement of their leaders to guide them through these precarious conditions (Dirani et al., 2020; Li et al., 2020). Researchers have also suggested that the leadership of an organization can substantially influence follower well-being (Fullagar and Kelloway, 2012; Rahimnia and Sharifirad, 2015). Influential leaders will encourage positive outcomes through their authentic engagement with team members in an organizational setting (Park et al., 2017). Conversely, leaders who exhibit inauthentic behavior and fail to create a supportive environment will adversely affect the well-being of team members (Bhandarker and Rai, 2019).

In an attempt to mitigate the challenges that modern organizations face, as well as enhance positive individual and organizational outcomes, an interest in positive leadership styles such as authentic leadership has been the focus of recent studies (Sidani and Rowe, 2018; Weiss et al., 2018). Because authentic leadership as an influential resource (Adil and Kamal, 2019; Adil et al., 2019) can promote the resources necessary for team members to confront challenging work demands, it may be an effective leadership approach under these conditions. The focus on authentic leadership during difficult times may be due to the positive characteristics such as resilience, confidence, optimism, transparency, ethical behavior, and future-oriented viewpoints that authentic leaders exhibit (Daraba et al., 2021). These qualities may affect employee and organizational outcomes positively.

Baykal (2020) posits that authentic leaders exhibit empowering qualities and can influence organizational support perceptions and organizational commitment positively. Additionally, perceived organizational support has been identified as a job resource (Hobfoll et al., 2018) and found to predict increased trust in the organization (Chen et al., 2005). Improved organizational trust is likely brought about by the perception of followers that the business is concerned about their welfare, appreciates their effort and contribution, and will do its best to meet their needs. Jaškevičiūtė et al. (2021) found a direct and an indirect relationship between trust in the organization and employee well-being. Trust in the workplace is recognized as an important job resource (Alcoba and Phinaitrup, 2021), as it can enhance motivation levels and foster employee well-being.

It is likely that employees who feel supported may experience a measure of benevolence that has been found to be a predictor of trust (Colquitt et al., 2007; Lapidot et al., 2007). If employees feel that they are treated with compassion and kind-heartedness it might cause them to experience psychological safety. The increased level of psychological safety could likely result in employees spending more time and energy on their work as opposed to devising strategies to find additional resources. Because employees feel supported and psychologically safe, they might reciprocate with increased levels of trust in the organization. Moreover, it can be argued that employees who feel supported and cared for could experience heightened levels of emotional well-being because they do not feel unsafe and without the necessary resources. Changes are that they will have more energy to focus on the task at hand and feel more motivated and engaged which points to components of psychological well-being. Lastly, because trust is relational in nature, employees who trust may reciprocate, as suggested by social exchange theory, resulting in enhanced positive teamwork and more harmonious interpersonal relations that could enhance social well-being.

According to Sun (2019) the way organizations treat their employees may influence their experience of support, which, will have an impact on employee attitudes. By integrating the arguments above, it is possible to argue that when authentic leadership enhances the employee perceived organizational support, they feel more trusting toward the organization because they are of the opinion that the organization has their best interest at heart and wants to see them develop and be successful, which eventually leads to their enhanced level of flourishing. The rationale for postulating that the sequential mediation of organizational support and trust in the organization in the relationship between authentic leadership and employee flourishing is thus plausible. We propose that employees who experience their leaders as authentic are likely to flourish in their jobs, firstly because they appreciate the support they receive, and secondly, they trust that the organization have their best interest at heart. Therefor it is desirable to suggest and test the sequential mediation chain so as to accurately understand how authentic leadership has an effect on flourishing. Flowing from this discussion, the following hypothesis was formulated:

**Hypothesis 4:** perceived authentic leadership is indirectly and positively related to flourishing, sequentially mediated through first organizational support, and then trust in the organization.

## Materials and methods

### Participants

This study focused on the managers employed at a prominent South African steel manufacturing organization. The target group consisted of 570 potential participants, and 314 of the candidates participated, culminating in a 55% response rate. Data collection was performed in 2020 by applying stratified random sampling.

The respondent characteristics were as follows: a total of 41.7% of the respondents were between 51 and 60 years of age. The majority of the participants (64.7%) stipulated that they functioned at the senior management level, while 19.1% specified that they had 11 to 20 years of service. A smaller portion of the participant population (24.2%) indicated that they had 11 to 20 years of experience in their current job.

### Measures

Participants were requested to complete a biographical information document together with the survey to appraise authentic leadership, organizational support, trust in the organization, and employee flourishing.

Employee perception of authentic leadership characteristics was measured by administering the Authentic Leadership Inventory (ALI; Neider and Schriesheim, 2011) to measure four subdimensions: self-awareness, internal moral perspective, balanced processing, and relational transparency. An example item is “My leader uses his/her core beliefs to make decisions”. The individual items (14 in total) were scored on a five-point Likert-type scale, ranging from 1 (*strongly disagree*) to 5 (*strongly agree*). Previous research in a South African setting confirmed a Cronbach’s alpha value of α = 0.93 (Stander et al., 2015) for the ALI. The ALI was successfully applied in a study by Crawford et al. (2020).

Organizational support was measured by using the seven-item related part of the Job Demands-Resources Scale (JDRS) instrument developed by Jackson and Rothmann (2005). An example item is “Does your work give you the feeling that you can achieve something?”. The items were appraised on a scale ranging from 1 (*never*) to 4 (*always*). The Cronbach’s alpha reliability coefficient was 0.88 (Rothmann et al., 2006). The Job Demands-Resources Scale was applied with success in a study by Cornelisse (2021).

One scale of the Workplace Trust Survey (WTS) (Ferres and Travaglione, 2003) was used to measure trust in the organization (11 items), which was scored by means of a seven-point Likert scale, ranging from 1 (*strongly disagree*) to 7 (*strongly agree*). An example item is “There is a widely held belief that my organization is moving forward for the better” (Ferres and Travaglione, 2003). A recent study that applied the WTS in a South African setting reported a composite reliability coefficient of.96 (Kleynhans et al., 2021b). Ramdas and Patrick (2019) administered the WTS successfully in their research.

The Flourishing-at-Work Scale – Short Form (FAWS-SF), consisting of 17 items in total, was employed to capture the frequency of specific symptoms experienced during the preceding month on a six-point scale, ranging between 1 (*never*) and 6 (*every day*). Psychological well-being (nine items, e.g., “How often did you get excited when you perform well on your job?”) reflects the frequency of psychological wellness. Emotional well-being (three items, e.g., “How often did you feel grateful?”) indicates the frequency of feelings of emotional wellness. Social well-being (five items, e.g., “How often did you feel that you really belong to your organization?”) represents the frequency of social wellness feelings. Previous research in a South Africa setting reported reliability values ranging between 0.77 and 0.89 (Rautenbach and Rothmann, 2017a). The FAWS-SF was applied with success in research conducted by Heyns et al. (2021).

Many survey designs state single single-method bias as a limitation. In this study, single-method bias was mitigated by limiting the number of items in the research questionnaire, applying different response set-ups (Podsakoff et al., 2012), and phrasing the questions clearly and concisely (Podsakoff et al., 2003). Additionally, participants were made aware of their anonymity, contributing to honest answers (Steenkamp et al., 2010). Finally, standardized questionnaires were utilized.

### Statistical analysis

Descriptive statistics and correlation analyses were computed to describe the data and the sample characteristics. As Cronbach’s alpha does not provide a reliable estimation of scale reliability when factor loadings differ (Raykov, 2009; Wang and Wang, 2020), we did not compute alpha coefficients, but preferred to compute omega reliability coefficients instead. A cut-off value of.70 for scale reliability, as proposed by Nunnally and Bernstein (1994), was used.

Latent variable modeling in Mplus 8.8 (Muthén and Muthén, 1998–2022) was used to test the measurement and structural models. The variables were considered continuous, and as there were no missing values, the mean-adjusted maximum likelihood (MLM), which is robust to the non-normality of data (Wang and Wang, 2020), was used as an estimator. Model fit was evaluated by means of the following indicators: the chi-square statistic (the test of absolute model fit), Tucker-Lewis index (TLI), comparative fit index (CFI), root mean square error of approximation (RMSEA), and standardized root mean residual (SRMR). TLI and CFI values with a minimum threshold of.90 are acceptable, although values of.95 or higher are preferred; RMSEA and SRMR values lower than.08 indicate a close fit between model and data (Wang and Wang, 2020). In addition, the Akaike information criterion (AIC), which is meaningful when estimating different models, and the Bayesian information criterion (BIC), which indicates model parsimony, were also used to appraise the fit of competing models. Lower AIC and BIC values point toward better fit (Wang and Wang, 2020).

For mediation analysis purposes, a serial multiple mediator model, as proposed by Hayes (2017), was employed using Hayes’s PROCESS macro as preferred regression-based path analytic technique (Hayes, 2017). To establish whether dependent variables were indirectly affected by independent variables via mediating variables, bootstrapping (5000 samples) was used to construct two-sided bias-corrected 95% confidence intervals (CIs) (Hayes, 2017). We could demonstrate mediation in the event that zero did not lie within the range of values represented by the upper and lower CIs.

## Results

### Testing the measurement model

To evaluate the distinctness of the measured variables, four competing measurement models were tested by means of confirmatory factor analysis. The first model (Model 1) was configured as the theory proposes: authentic leadership was specified as a second-order latent variable, consisting of four first-order latent variables that were allowed to correlate, namely, self-awareness (three items), internal moral perspective (four items), balanced processing (four items), and relational transparency (three items). Trust in the organization (alternatively referred to as organizational trust) was measured by 11 directly observed items, and organizational support was measured by seven directly observed variables. Flourishing was also specified as a second-order latent variable, consisting of three first-order latent variables that were allowed to correlate, namely, emotional well-being (three items), psychological well-being (nine items), and social well-being (five items). The results indicated an acceptable fit to the data (χ^2^ = 1826.53, *df* = 1111, *p* < 0.001; CFI = 0.911; TLI = 0.906; RMSEA = 0.045, *p* > 0.983 [0.042,0.049]; SRMR = 0.055).

Three alternative models were specified. For Model 2, all factors were expressed similarly to those in the first model, except for authentic leadership, which was now specified as a single latent factor measured by 14 directly observed variables. Model 2 (χ^2^ = 1840.20, *df* = 1115, *p* < 0.001; CFI = 0.910; TLI = 0.905; RMSEA = 0.046, *p* = 0.978 [0.042,0.049]; SRMR = 0.055) offered an acceptable, yet slightly poorer, fit to the data compared to Model 1.

For Model 3, all factors were expressed similarly to those in Model 1, except for employee flourishing, which was now specified as a single latent factor measured by 17 directly observed variables. This model (Model 3) offered a clearly poorer fit to the data than the first two models.

We also tested a final competing measurement model (Model 4), where all constructs were measured by directly observed variables only. This model rendered the least acceptable fit to the data when compared to the first three alternatives. Table 1 presents the fit statistics for the competing measurement models.

**TABLE 1:** Fit statistics for the competing measurement models.

M	Chi-square	*df*	TLI	CFI	RMSEA	SRMR	AIC	BIC
1	1826.53*	1111	0.906	0.911	0.045	0.055	37078.10	37689.25
2	1840.20*	1115	0.905	0.910	0.046	0.055	37086.14	37682.30
3	1997.18*	1114	0.884	0.890	0.050	0.059	37260.86	37860.77
4	2010.76*	1118	0.883	0.889	0.050	0.059	37268.90	37853.80

df, degrees of freedom; TLI, Tucker-Lewis index; CFI, comparative fit index; RMSEA, root square error of approximation; SRMR, standardized root mean square residual, *p < 0.01.

Comparison of the fit indices indicated that Model 1 fitted the data best and was also the model that was closest to what the theory proposes. The standardized regression coefficients were all statistically significant (*p* < 0.01), and all items loaded on their respective constructs as expected, with values ranging from 0.404 to 0.987. Table 2 presents the correlation matrix for the latent variables, including their corresponding reliability coefficients.

**TABLE 2:** Descriptive statistics, reliabilities, and correlations of the scales.

Variable	Mean	*SD*	*p*	1	2	3
1 Authentic leadership	3.61	0.65	0.98	–	–	–
2 Organizational support	3.13	0.48	0.78	0.64		–
3 Trust in the organization	3.65	1.26	0.93	0.48	0.49	–
4 Flourishing	4.45	0.77	0.93	0.45	0.74	0.79

All correlations were statistically significant (p < 0.01). Parameters for the correlation coefficients were considered as a small effect when r ≥ 0.10, medium effect when r ≥ 0.30, and large effect when r ≥ 0.50 (Cohen, 1998).

The composite reliability of all the scales was above the cut-off point of.70. Table 2, furthermore, shows that all correlations were statistically significant. Correlations of large effect were recorded for the relationships between authentic leadership and organizational support, between organizational support and employee flourishing, and between trust in the organization and employee flourishing. Correlations approaching a large effect were recorded for the relationships between authentic leadership and trust in the organization, between authentic leadership and employee flourishing, and between organizational support and trust in the organization.

### Testing structural models

The structural model was tested based on the results of the ideal measurement model. The results indicated a good fit of the re-estimated model to the data: χ^2^ = 1786.91, *df* = 1110, *p* < 0.01; RMSEA = 0.044 (90% CI:0.040,0.048); CFI = 0.916; TLI = 0.911; SRMR = 0.055. Although the chi-square test of model fit was statistically significant, the RMSEA and SRMR were below.08, and the TLI and CFI values were acceptable, with the results above the.90 criteria. Table 3 shows the standardized coefficients estimated by Mplus for the structural model. From Table 3, it is evident that organizational support and trust in the organization positively and statistically significantly predicted employee flourishing. Hypothesis 1a and b were supported, but 1c could not be accepted.

**TABLE 3:** Standardized regression coefficients of authentic leadership, organizational support, and organizational trust, in predicting employee flourishing.

Variable	Estimate	SE	Estimate/SE	*p*
**Flourishing as predicted by**				
Authentic leadership	–0.46	0.043	–1.067	0.286
organizational support	0.580	0.041	14.300	0.000*
organizational trust	0.471	0.042	11.230	0.000*
				

*p< 0.01.

### Mediating effects

Due to the significant regression relationships from authentic leadership to organizational support and trust in the organization, respectively, as well as from organizational support and trust in the organization to employee flourishing, mediation analyses were performed to consider the roles of organizational support and trust in the organization as potential underlying mechanisms through which authentic leadership might indirectly affect employee flourishing.

Through the Hayes (2017) procedure, a serial multiple mediation model was tested. Employing 5000 bootstrap samples, bias-corrected 95% CIs were estimated. The unstandardized regression coefficients of the variables were used (Montoya and Hayes, 2017).

The mediation model simultaneously tested two alternative single-mediator pathways, as well as a two-mediator sequential pattern, to consider all possible alternative ways in which the predictor variables might influence employee flourishing. In addition, two demographic aspects – job category and employees’ years of service in their current positions – were controlled for in each instance.

Firstly, the model tested whether the effect of an authentic leadership style on employee flourishing was mediated through the provisioning of organizational support alone. The indirect effect was significant (β = 0.2053, 95% CI = [0.1401 to.2760]), thereby indicating that organizational support served as an underlying mechanism through which an authentic leadership style promoted employee flourishing. Hypothesis 2 was accepted.

Secondly, the model tested whether the effect of an authentic leadership style on employee flourishing could be explained by trust in the organization as a facilitative mechanism, independent of the degree of organizational support provided to the employees. This indirect effect was also positive and statistically significant (β = 0.1813, 95% CI = [0.1177,0.2519]) and confirmed that an authentic leadership style would enhance employees’ trust in the organization, which, in turn, would promote higher levels of employee flourishing at work. Hypothesis 3 was supported.

Finally, the indirect effect of the two-mediator sequential pattern was also significant, as indicated by the fact the 95% CI did not include zero (β = 0.0624, 95% CI = [0.0328,0.0994]). This finding was consistent with the hypothesis that organizational support enhanced employees’ trust in the organization, which, in turn, was the more proximal predictor of employee flourishing. Hypothesis 4 was accepted.

**FIGURE 1:**
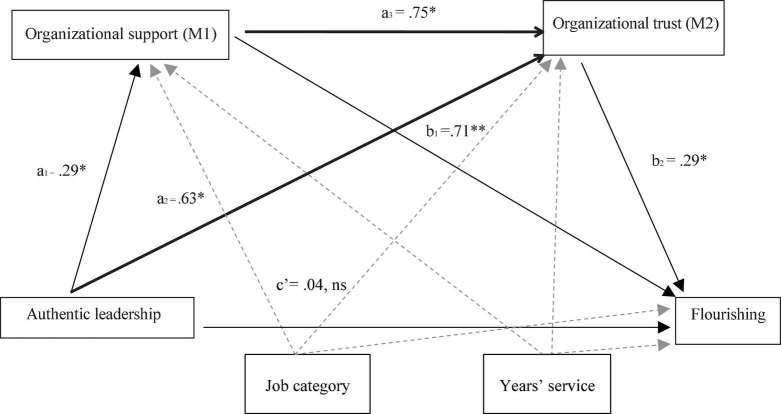
Two-mediator sequential model depicting the relationship between authentic leadership and flourishing as mediated by organizational support and trust, controlling for job category, and years’ service in current position. Asterisks indicate significant coefficients (*p < 0.05).

Figure 1 below displays the full model, with unstandardized β weights for the path coefficients.

In summary, the data were consistent with the claim that authentic leadership had no direct effect on employee flourishing. Instead, authentic leadership had a substantial indirect, positive effect on employee flourishing through organizational support and trust, firstly, as underlying mechanisms that operated simultaneously, but independently of each other, as well as serially through organizational support, which enhanced trust in the organization, in turn promoting employee flourishing. The full model, including mediators and controlling for the covariates, accounted for 42% of the variance in employee flourishing.

## Discussion

The job demands-resources (JDR) framework has established job resources as critical elements contributing to employees’ wellness. According to Bakker and Demerouti (2017), a lack of resources can lead to a health impairment process, resulting in poor well-being. In contrast, organizational resources can lead to a motivational process with positive outcomes (Schaufeli and Taris, 2014). In this research in a manufacturing context, we investigated how authentic leadership, trust in the organization, and organizational support, as potential job resources, influenced employee well-being (outcome) during uncertainty (COVID-19).

While controlling for job category and years of service in the current position, our first findings indicated that all the correlations between constructs were statistically significant, while correlations of large effect were recorded for the relationships between authentic leadership and organizational support, between organizational support and trust, and between trust and employee flourishing. All the other correlations were of medium effect. Authentic leadership was seen as a direct antecedent of organizational support. According to the literature, by being authentic, that is, being genuine and trustworthy, leaders establish constructive organizational conditions (Avolio and Gardner, 2005) and create trust (Gardner et al., 2005; Stander et al., 2015; Coxen et al., 2016). Employees who experienced their leader as authentic were likely to perceive the organization as supporting them. The positive relationship between organizational support and trust strengthens the conclusion reached by Zeng et al. (2020) that support as a resource generates positive emotions – in this study, trust – among employees. Additionally, Kleynhans et al. (2021a) mention that leaders who are open and allow employees to get to know them instill trust in the organization.

The positive relationship between organizational support and employee flourishing corroborates previous research (Kurtessis et al., 2017; Imran et al., 2020; Ho and Chan, 2022) and, at the same time, illustrates the importance of healthy work environments in uncertain times. In terms of the research context of this study, the particular manufacturing organization had been confronted with tough business challenges even before COVID-19. Having been exposed to support before could have softened the impact of the pandemic and strengthened employee trust in the organization. Employees could have trusted that the organization had demonstrated resilience in confronting business challenges in the past, while caring for its people. Therefore, they could have trusted that the same would happen in future.

Furthermore, from the results, it was evident that a strong relationship existed between authentic leadership, organizational support, trust in the organization, and employee flourishing. This supports organizations investing in interventions that will enhance trust and create psychologically safe environments where employees feel that the organization has their best interests at heart. These findings imply that leaders positively influence employees when they illustrate personal insight and active listening and consider employees’ opinions and ideas, while being transparent in their interactions. Demonstrating these behaviors will create a feeling of psychological safety and promote trust in the organization. In this study, the support and trust led to a positive outcome: the flourishing of employees. This outcome is similar to previous research that established that authentic behavior had a profound influence on creating supportive workspaces and trust (Alkaabi, 2018; Malik et al., 2020), as well as the well-being of employees (Kernis, 2003; Ilies et al., 2005; Rautenbach and Rothmann, 2017b; Kerns, 2018; Agarwal, 2021; Nair et al., 2021), directly or indirectly. What makes these results more significant is that during times of COVID-19, leaders had less time to interact on a one-to-one basis or to demonstrate support and care. Having such a positive effect on employees in uncertain times reinforce the value of being an authentic leader. Without having empirical evidence, one may reason those authentic leaders possibly have an even more substantial influence on trust and feelings of support in uncertain times compared to normal circumstances. Chully et al. (2022) found that in a pandemic world, authentic leaders need to ensure that employees are comfortable sharing their opinions while the leaders support them and build a flexible and innovative culture that can endure the disruptive external environment. To sustain trust in circumstances such as a pandemic, leaders should remain closely connected to employees and demonstrate actions to enhance the strength of the authentic relationship (Ahern and Loh, 2020).

Significant regression relationships from authentic leadership to organizational support and trust in the organization, respectively, and from organizational support to employee flourishing were found. The direct effect of organizational support and trust in the organization on employee flourishing was positive and significant. Authentic leadership positively and statistically significantly predicted employee flourishing via organizational support and trust in the organization. In addition, from the results, it was evident that trust in the organization and organizational support significantly predicted employee flourishing. Being transparent and objective and behaving with integrity, authentic leaders contributed to developing healthy work environments with open, trustful relationships between the employees and the organization. The findings support Baran and Woznyj (2020) and Rath et al. (2021), postulating that employees depend on leaders for support and guidance. The present results, moreover, revealed that employees’ experiences of organizational support contributed to trust in the organization. Ultimately, this positive experience of support and trust influenced employees’ psychological, emotional, and social well-being and benefitted employees and the organization (Schotanus-Dijkstra et al., 2016). The results support Jaškevičiūtė (2021), who found that trust in the organization and trust in its leaders were positively associated with employee well-being.

Mediation analyses were performed to consider the roles of organizational support and trust in the organization as potential underlying mechanisms through which authentic leadership might indirectly affect employee flourishing. The study supports the mediating role that organizational support can play in the relationship between perceived authentic leadership and trust. This implies that, when individuals perceive their leaders as authentic, organizational support can be seen as a facilitative process that translates the perceived authenticity into a higher level of trust in the organization. This result is in line with the social exchange theory. Its basic principle is that relations are formed based on cost-benefit analysis, and people tend to duplicate behavior rewarded in the past (Blau, 1964). In this study, a good relationship between leaders and employees created a constructive climate, influencing the level of trust in the organization. It was then worth it for the employees to trust in the organization again in future (Dirks and Ferrin, 2001).

The results showed that trust played a mediating role in the relationship between organizational support and employee flourishing. Perceived organizational support improved trust in the organization, while trust strengthened the relationship between organizational support and employee flourishing. The results support the notion that trust plays a key role in enhancing employee wellness in uncertain times. Support and trust will enhance the experience of flourishing, and according to the literature, flourishing employees manage uncertainty better (Schotanus-Dijkstra et al., 2016). This result confirms the importance of protecting and maintaining the well-being of employees in challenging times (Deloitte, 2020) by practicing positive leadership, providing support, and enhancing trust in the organization.

Testing for serial mediation, authentic leadership positively and statistically significantly predicted employee flourishing via the directional impact of organizational support on trust in the organization. The level of trust will increase when employees perceive the organization as investing time and energy in meeting their needs, contributing to a higher level of trust in the organization. Organizational support on trust in the organization played a combined facilitating role, where authentic leadership indirectly predicted employee flourishing. Organizational support predicted trust in the organization. To be able to trust, employees needed evidence of previous positive experiences. Experiencing a healthy organizational climate, even in times of extreme demands, would, therefore, enhance employees’ confidence in the organization, trusting that the organization would prioritize their needs and demands. Based on the principle of reciprocity, Sun (2019) mentions that how organizations treat their employees can influence perceived support, which, in turn, will have an impact on employee attitudes and behavior – in this case, trust.

This study provided empirical evidence that authentic leadership promoted employees’ feelings that the organization supported them, while creating higher levels of trust in the organization. Employees’ perceptions of their leaders’ authenticity stimulated employee wellness through organizational support and trust in the organization. Previous research suggested that an uncertain, insecure work context harmed employee well-being (Standing, 2011; Utzet et al., 2020; Bakker and Van Wingerden, 2021). The findings in this study indicated that authentic leadership, organizational support, and trust in the organization could counterbalance the harm of uncertain work contexts.

These results are consistent with other research reporting that the role of leaders is imperative in challenging business environments (Baran and Woznyj, 2020; Rath et al., 2021). Wang et al. (2014) view authentic leadership as a positive approach that can be described as transparent, moral, and genuine, making a valuable contribution during precariousness. More specifically, leaders must create a positive work environment in which organizational support and trust in the organization are endorsed and encouraged. Employees who feel that the company offers opportunities for growth, development, and achievement will trust that the organization considers their needs and best interests, while treating them fairly. One can expect that such leadership behavior will influence employees’ wellness and contribute to organizational outcomes such as performance, extra-role behavior, and the retention of high-potential employees.

### Managerial implications

Some of the significant challenges facing organizations in the next few years are retention of talented people (“the great resignation”), being digital fit, managing ambiguity, fostering learning agility and change, and enhancing the wellness of their employees. In times of uncertainty and volatility, employees must trust their organization to implement initiatives that can possibly alleviate the negative effect of internal and external forces on the organization. Our results support the notion that organizational support and trust in the organization can be considered essential explanatory mechanisms in making it clear how positive perceptions of leadership may translate into employee flourishing in challenging times. This study confirmed that when employees experienced their leaders as being authentic, being self-aware, having a moral perspective, and displaying objectivity and transparency, would enhance feelings of trust and being supported by the organization. Ultimately, this would improve employee flourishing. From a JDR perspective, authentic leadership, organizational support, and trust in the organization are resources that should be nurtured to limit the influence of an imbalance between resources and demands, especially in times of uncertainty.

Organizations should invest time and energy in developing authentic leaders. Authentic leadership is a valuable resource that has an add-on effect: in this research, enhanced trust and experiences of support. One can assume that the only constant will be a continuous change in the future. The impact of change, uncertainty, complexity, and ambiguousness results in discomfort, adding a demand on the consumption of energy. Managers should focus on spending time with their people to get to know them, optimize their strengths, and create a psychologically safe environment by sharing information, being open, developing people, and showing concern for wellness. This support and care will inform employees that their leaders are genuinely concerned about them as human beings and not only as production factors. Support will lead to positive expectations regarding the leaders’ intentions and the organization. When employees experience trust in the organization and its leaders, it will soften the impact of a demanding business environment, creating feelings of “being in this together”.

Healthy positive relationships between employees and leaders will form the cornerstone of a positive work climate that is conducive to building trust and enhancing employees’ wellness. Experiencing a positive organizational environment and trusting the intentions of the organization will strengthen employees’ willingness to deal with challenges and cope with a volatile, uncertain, ambiguous, and complex business environment. Leaders must act as role models, authentically living their values. Leaders should accept that specific external threats are beyond their control. Still, leadership development and the creation of a trustful climate where employees feel safe to optimize their potential are within the control of the organization.

### Contribution, limitations of the study, and recommendations for future studies

This study contributed to the theory by suggesting that authentic leadership directly created supportive working conditions and created trust. The positive association of authentic leadership with support and trust and the indirect effect on employee flourishing through the two variables mentioned highlighted the potential value of authentic leadership in the broader South African business context and, specifically, in the manufacturing industry. The findings of this study may also be extended to other industries that find themselves in an unstable business environment. This study attempted to add value by clarifying and providing understanding of the positive role of job resources in contributing to employees’ wellness in times of exposure to hostile forces.

This research was not without limitations. Firstly, using a cross-sectional design did not allow investigation of the causal relationships between the variables. Secondly, the use of self-report information could have influenced the accuracy of results. Nevertheless, an effort was made to limit possible bias and ensure trustworthy responses. The respondents were assured that participation was voluntary and that they could withdraw at any time, that there were no right or wrong answers, and that the data would only be used in an aggregated format. A third limitation related to the sample. The study focused on only one company, and it might, thus, be interesting to determine whether the same results would be achieved in the industry or manufacturing in general. The current study focused on management levels within the manufacturing organization. Future studies could include all levels of employees. Similar future studies should consider a longitudinal approach to interrogate the relation between authentic leadership, trust in the organization, and employee flourishing over time to determine whether changes in the business context will affect the findings of this study. The vital role of organizational resources was highlighted in this research. The role of personal resources in times of uncertainty should be explored in the future. Currently, there is a qualitative and quantitative gap in research on the role of job resources compared to research on organizational or job aids. Exploring how the availability of personal resources might influence the way in which job resources and demands are experienced in challenging times could add value to the field of organizational psychology.

## Conclusion

Based on our results and discussion, this study, thus, suggested that authentic leadership indirectly affected trust in the organization and feelings of support. Concurrently, the last two directly influenced employee flourishing. Employee wellness will be one of the most critical challenges for organizations and human capital practitioners in the next few years. The results indicated the value that leaders with insight, acting without personal biases, sharing information transparently, and building healthy relationships contributed to emotional wellness, with an expected positive impact on organizational health. From a JDR perspective, leadership, support, and trust in the organization resources ignited a motivational process that could counterbalance job demands in times of ambiguity. The results suggested that, despite operating in a turbulent context, endorsing an authentic leadership style could have beneficial individual consequences.

## Data availability statement

The raw data supporting the conclusions of this article will be made available by the authors, without undue reservation.

## Ethics statement

This study involving human participants were reviewed and approved by the Economic and Management Sciences Research Ethics Committee (EMS-REC) of the North-West University, Potchefstroom, South Africa (ethics number: NWU-00609-20-A4). The participants provided their written informed consent to participate in this study.

## Author contributions

MH and MS made contributions regarding the conceptualization, review, and editing of the study. MH performed the statistical analysis. All the authors contributed to the article and approved the submitted version.
